# Decision-making, therapy, and outcome in lateral compression fractures of the pelvis – analysis of a single center treatment

**DOI:** 10.1186/s12891-019-2583-3

**Published:** 2019-05-15

**Authors:** J. Zwingmann, H. Eberbach, P. C. Strohm, N. P. Südkamp, J. Lauritsen, H. Schmal

**Affiliations:** 1 Department of Orthopaedics and Trauma Surgery; Faculty of Medicine, Medical Center - Albert-Ludwigs-University of Freiburg, Freiburg, Germany; 2 Department of Orthopaedics and Traumatology, Odense University Hospital and Department of Clinical Research, University of Southern Denmark, Sdr. Boulevard 29, 5000 Odense C, Denmark; 3 Department of Orthopaedics and Traumatology, Sozialstiftung Bamberg, Klinikum am Bruderwald, Bamberg, Germany

**Keywords:** Pelvic fracture, Lateral compression, Treatment, Register, Logistic regression, Operative, Non-operative, Follow-up

## Abstract

**Background:**

Pelvic lateral compression fractures are the most stable of the unstable fractures. Therefore, decision making regarding operative or non-operative therapy is still a matter of debate.

**Methods:**

Factors, influencing decision making for therapy, were explored based on prospectively collected register data of a single Level-1 trauma center. The analysis included epidemiological records such as age and gender, and injury characterizing parameters such as degree of displacement and the Injury Severity Score (ISS). In-hospital mortality and complications served as short-term outcome variables. After matching for relevant confounders, long-term results were compared between operatively and non-operatively treated patients, evaluating the Merle d’Aubigne and the EQ. 5D-3 L scores.

**Results:**

Over an 11-year period (2004–14), 134 patients suffered from lateral compression fractures out of 567 pelvic fractures (33%). After excluding patients with clear indications for operation (complex pelvic fractures and pubic symphysis ruptures) and pediatric fractures, 114 patients could be included in the analysis. Sixty-one patients were treated conservatively (54%), 53 with an operation (46%). The operated patients were younger (43.7 vs 58.3 years), had higher ISS (19.9 vs 15.5 points) and fracture displacements (2.3 vs 4.9 mm) (*p* < 0.001 for all). The length of hospital stay was shorter in the conservatively treated group (12.7 vs 17.3 days, *p* < 0.02). Although the types of complications were different, the incidence was not. The mortality was less in the operated group (1.9% vs. 6.6%), however, a logistic regression analysis showed that only the ISS was an independent risk factor, but not the type of therapy. Merle d’Aubigne and EQ. 5D-3 L scores were not different in the matched cohorts.

**Conclusion:**

Decision-making for operative therapy was favored in severely injured young patients with high displacement. However, short- and long-term outcomes showed no difference between operatively and non-operatively treated patients.

**Trial registration:**

DRKS, no. 00000488. Registered 14th July 2010 - Retrospectively registered

## Background

Pelvic fractures have been associated with a decreased quality of life and an overall high mortality. Historically, pelvic fractures have been classified based on pelvic stability and the mechanism of injury as proposed by Pennal and modified by Tile. Basically, their classification discriminates stable A-type injuries without biomechanical interruption of upper and lower body parts, B-type injuries with rotational instability, and C-type fractures with an additional vertical shear component. This is the classification adapted by AO/OTA. There is a wide agreement that patients with type A fractures are mainly treated conservatively, while those classified as C-fractures are frequently operated [1, 2].

Lateral compression fractures of the pelvis (Young Burgess LC, OTA B1, B2) are the most frequent pelvic injury types [3] and biomechanically the most stable of the unstable fracture types. While a type 1 lateral compression fracture (LC1) refers to crush or ‘buckle fractures’ of the sacrum according to the definition provided by Young et al. [4], the fracture line of type 2 injuries (LC2) involves the sacroiliac joint and some of the posteriorly stabilizing ligaments. In lateral compression injuries type 3 (LC3) which Young et al. [4] also called ‘windswept pelvis’ are parts of both iliosacral joints destroyed why these fractures may be considered rotationally and vertically unstable and therefore are not of interest for this article. All lateral compression injuries possess typical fracture patterns of the obturator ring. Although it was previously pointed out that therapy should consider whether the fracture line is limited to the anterior part of the sacrum or extends to the posterior structures [1], this feature is not decisive for the classification of Young and Burgess where the category LC1 covers minimal impactions of the anterior sacrum and comminuted fractures also involving the posterior cortex [5]. While a displacement of 1 cm is generally considered to be critical regarding stability and requires operative stabilization, it still remains unclear to which degree an operation is preferable when displacement is less [6–8].

Although the relevance of operative therapy for type B and C pelvic fracture has recently been highlighted [9], uncertainty remains regarding the role for stabilizing the subgroup of lateral compression fractures summarized as B2-type to prevent future displacement resulting in later deformity [10, 11]. This is evidenced by a recent article by Khoury et al. in which it is stated that “the indications for non-operative and operative treatment of LC fractures remain unclear” [12]. Few data correlate radiographic displacement with functional outcome and some exist demonstrating good outcomes with non-operative treatment; this further clouds the decision process [13, 14].

A new study found no evidence that surgical stabilization of certain LC1 and LC2 pelvic fractures improves patient’s pain, decreases their need for analgesics, and improves time to mobilization [15]. Another study looked into how surgeons agree or disagree on whether there is an indication for LC1 and LC2 stabilization and showed that only 9 of the 27 cases (33%) had substantial agreement [16]. The fact that the lateral compression fracture is the most common injury of the pelvic ring emphasizes the need for clear guidelines to identify patients, who will likely benefit from an operative treatment [1, 2].

Therefore, a single center subgroup of the German Pelvic Trauma Registry is used for a study comparing the short- and long-term outcome of patients suffering from lateral compression fractures (B2). Besides grouping of treatment modality, factors characterizing the fracture and patient’s epidemiology are analyzed. Short-term outcome parameters are mortality, general complications and problems associated with the operation. Long-term parameters are quality of life (EQ. 5D-3 L Score) and the functional Merle d’Aubigne Score. The hypothesis for this comparison of operative and non-operative treatment was that there are determinants for decision-making, however, no differences for outcome measures were expected.

## Methods

### Pelvic trauma registry initiative

Data used for this study was collected within the Pelvic Trauma Registry Initiative, prospectively collecting records from patients with blunt and penetrating pelvic fractures [17–21]. Although this register includes data from 29 participating university hospitals and level I trauma centers, the presented analysis focusses on a single center. As described previously [22], the participants are required to register all treated cases in a primary electronic Case Report Form (eCRF), which are approved on a yearly basis. Documentation guidelines are supervised by a steering group and communicated during regular meetings twice a year. Data anonymity is guaranteed for the patient and participating hospital. From 2004, a secured internet interface hosted by a professional service provider (MEMDoc, Swiss medical Registries and Data Linkage, University of Bern, Switzerland) facilitated data management including processing and plausibility checks. Follow-ups were registered in a secondary eCRF of the same register. Data acquisition and analysis were done in accordance with ethical guidelines and approved by our institutional review board (no. 89/09). The trial was registered at the German Clinical Trials Register (no. 00000488), which also includes the follow-up.

### Analyzed parameters

All selected items were exported from the original data sets into a Microsoft Excel (Microsoft Corp, Redmond, WA) document for evaluation and statistical analysis. The following items were included: age (calculated), gender, Injury Severity Score (ISS), fracture type according to Tile’s classification [2], maximal displacement (calculated), length of hospital stay (calculated), type of treatment (operative or non-operative), complications, mortality, and carried out operative stabilization. The primary outcomes of the study complications and mortality were only registered during the primary hospital stay. Complications were summarized from general and intraoperative complications as previously described [22]. However, all patients with registered intraoperative complications had also general complications. Experienced orthopedic/trauma surgeons classified all fractures. Moreover, doubtful cases were discussed in regular meetings conducted by the working group to minimize inter-observer bias. In accordance with register guidelines, we defined pelvic injuries presenting major visceral, neurovascular or soft tissue injuries as complex pelvic injuries [23, 24]. The maximal displacement concerns only the posterior pelvic ring, displacement of the anterior fracture component (pubic rami) was neither documented nor considered relevant. The displacement was determined by measuring the maximal distance between fracture components in the different slices of the 2-dimensionally reconstructed CT scan, which is considered as a standard diagnostic procedure for the registry entry. Patient’s functional outcome was evaluated by the Merle d’Aubigne Score. This score is understandable, simple to administer, measures pain, gait and mobility of the hip, and ranges between 0 (worst) and 18 (best) [25]. Quality of life was assessed using the EuroQol 5D-3 L questionnaire [26]. Both scores used for follow-up were self-assessed (patient related outcome measure). For follow-up, patients were matched for age (10-years intervals) and ISS (10-point intervals) to reduce diversity of the 2 populations.

### Eligibility criteria

All pelvic fractures in the register treated between Jan 2004 and December 2014 were included in this analysis. All data sets were complete regarding the analyzed items. To extract the relevant type B2 fractures, patients with complex fractures, disruption of the pubic symphysis, and pediatric fractures (≤14 years) were excluded.

### Decision-making

Usually, an operation of lateral compression fractures was recommended when the anterior component was combined with a complete posterior fracture with a displacement > 5 mm [27]. Furthermore, sufficient mobilization should be possible within the first week. However, the indication for operative or conservative treatment included the individual consultant attitude and patient’s preference.

### Statistics

Bi-variate analysis for comparison of differently treated patients and the matched subgroups was used to assess the possible confounding variables. Distribution was analyzed using the Kolmogorov–Smirnov test. Normally distributed numeric data sets were compared using the unpaired Student’s t–test. The Mann-Whitney U test was applied comparing all ordinal variables such as scores; incidences were compared using the chi square test. Our data were primarily arranged using WinStat 2009 (Bad Krozingen, Germany) for Microsoft Excel (Microsoft Corp, Redmond, WA). Final statistical testing was done using STATA, version 14 (StataCorp LLC, College Station, TX, USA). Variables differing between groups were considered as potential confounders and included in the final multivariable logistic regression model. The odds ratios (OR) and corresponding 95% confidence intervals (CIs) were calculated for both mortality and complications. Significance level was set at alpha = 0.05 for all comparisons.

## Results

### Patient selection

During the inclusion period from January 1st, 2004 until December 31st, 2014 in total 567 cases with pelvic fractures treated at the Freiburg University Hospital, a Level-1 trauma center, were registered. After exclusion of isolated acetabular fractures or their combinations with pelvic fractures, the classification of the remaining 396 cases showed that the unstable fracture types (B and C) prevailed by far with a majority of the different B-types (Table 1).

**Table 1:** Overview about classification details

Tile types	total number	percentage
A1	20	5.1%
A2	46	11.6%
A3	6	1.5%
B1	49	12.4%
B2	134	33.4%
B3	28	7.1%
C1	64	16.2%
C2	30	7.6%
C3	19	4.7%
Total	396	100%

**Fig. 1:**
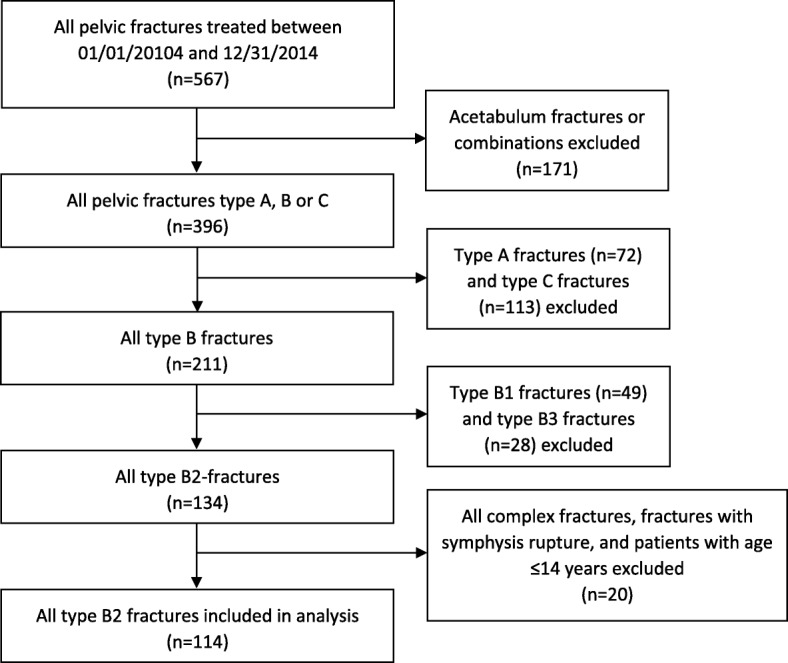
Patient selection

**Fig. 2:**
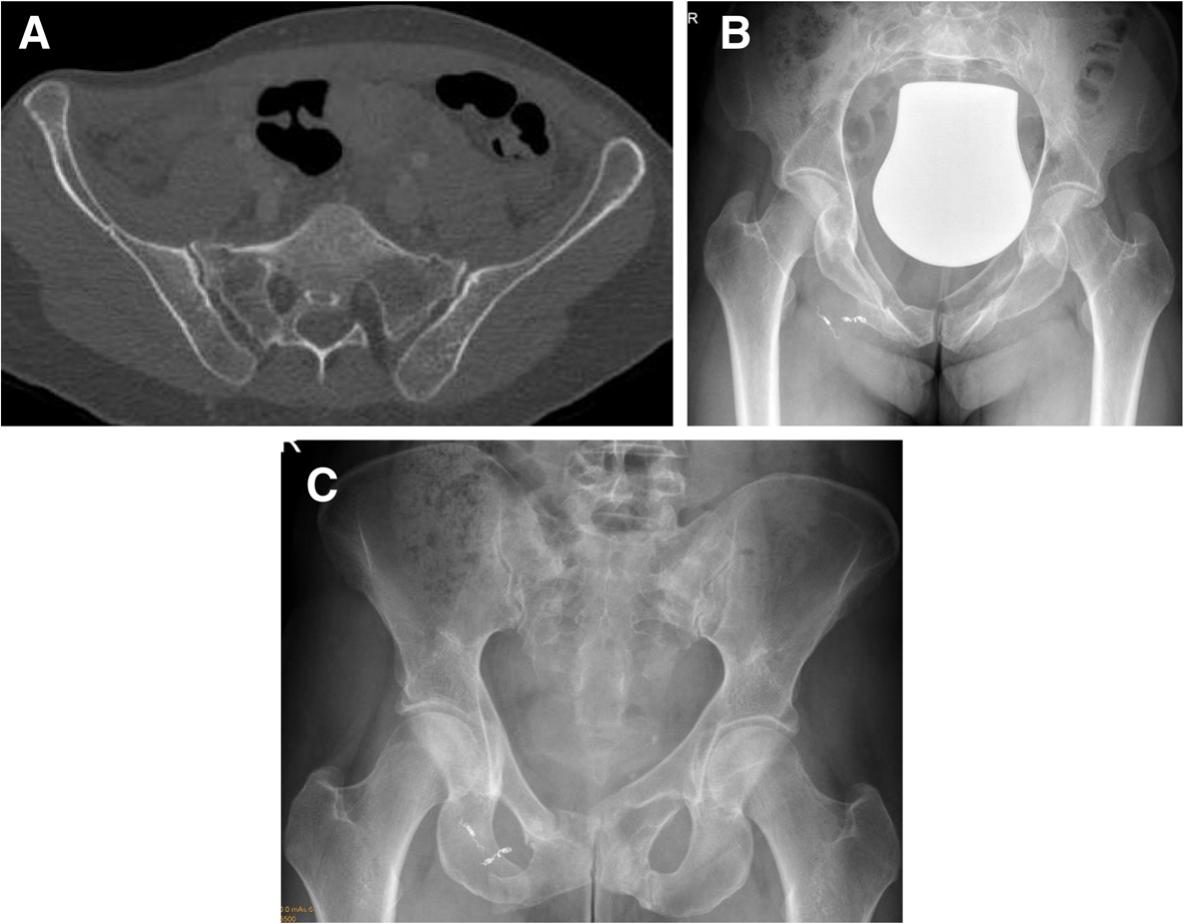
Clinical example for non-operative therapy of a lateral compression fracture. **a** shows the initial CT-scanning with sacral fractures on both sides. **b** shows the pubic rami fractures and the coils after embolization. **c** shows consolidation after 6 weeks. The patient presented with full weight bearing and without pain

**Fig. 3:**
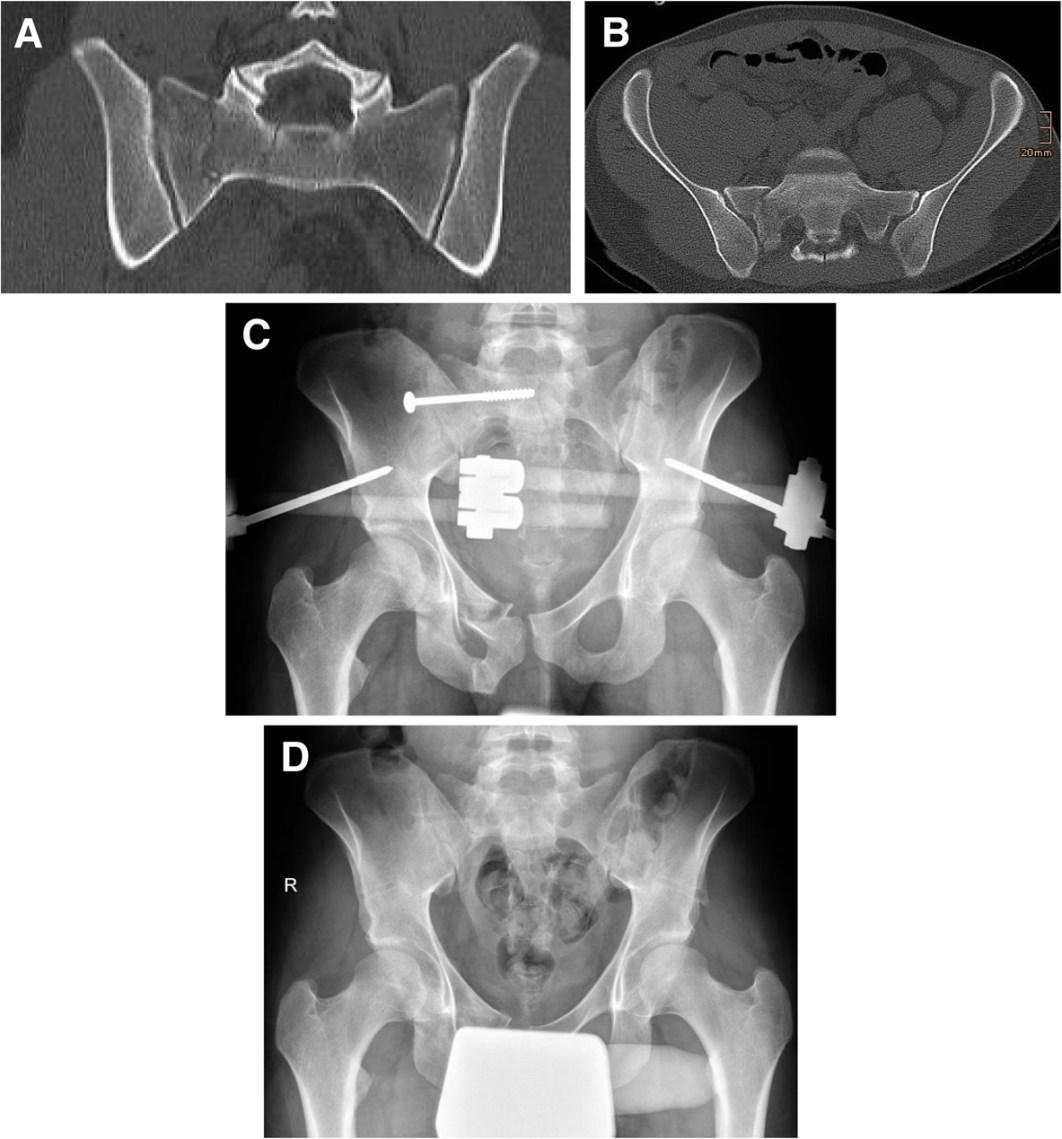
Clinical example for operative therapy of a lateral compression fracture. **a** and **b** show the initial CT-scanning with a sacral fracture on the right side. **c** shows the fixed situation after implantation of an ilio-sacral screw and anterior external fixation. **d** shows consolidation after implant removal 6 months following injury. The patient presented with full weight bearing and without pain

134 (33%) patients suffered from a lateral compression fracture (type B2). For the comparison of operative and non-operative therapy, patients with complex fractures (include injury of internal organs and open fractures), and with disruptions of the symphysis were excluded, because these cases usually require an operative intervention. Furthermore, pediatric fractures were excluded, because prognosis and complications differ from adult fractures as recently shown [21]. The final analysis was based on the remaining 114 type B2 fractures. Figure 1 represents the summary of the patient selection process. Figures 2 and 3 provide examples to illustrate the possible treatment success after non-operative (Fig. 2) and operative therapy (Fig. 3). However, operative treatment was associated with two interventions, implantation and implant removal.

### Epidemiology

Table 2 provides an overview about epidemiological characteristics and treatment data of the 114 fractures, which were included in the final analysis. The portions of operatively and non-operatively treated patients were almost leveled out, however, both groups substantially differed in all registered parameters. Summarizing, the younger, more severely injured patients with a higher displacement were significantly more likely operated. The numbers reflect that this group is in the majority male. Furthermore, operative treatment was associated with a longer length of stay in the hospital.

**Table 2:** Overview about the epidemiological characteristics between non-operatively and operatively treated patients with lateral compression fractures of the pelvis

	non-operative	operative	P
Patients (n)	61	53	
Age (years)	58.3 ± 23.1	43.7 ± 21.3	< 0.001^b^
Gender (male/female)	26/35	34/19	0.02167^a^
ISS	15.5 ± 2.3	19.9 ± 2.4	< 0.001^c^
Maximal displacement (mm)	2.36 ± 1.58	4.87 ± 3.33	< 0.001^d^
Length of hospital stay (days)	12.7 ± 11.5	17.3 ± 7.3	< 0.001^d^

*ISS* Injury Severity Score.

^a^chi square test; ^b^T-test; ^c^U-test (Mann-Whitney); ^d^Aspin-Welch T-test

Since fracture displacement is an obvious criterium for the kind of treatment, this data was further analyzed using median and percentiles. Hereby, the displacement of the pubic rami or obturator ring (anterior pelvis) is not considered, because this is not separately registered in the database following a consensus decision of the documenting institutions referring to the limited importance. Although the mean general displacement was higher in the operated group as shown in Table 2, the 90% percentile of the non-operatively treated patient (4.8 mm) was higher than the median of displacement in the operated patients (4 mm). Only 25% of the operatively treated patients had a displacement larger than 5 mm and 10% a higher displacement (> 8.6 mm) than the conservatively treated patients. This data illustrates a large overlap between the two groups.

### Mortality

The mortality was less in the operated group (1.9% vs. 6.6% or *n* = 1 vs. *n* = 4), but the difference did not reach statistical significance (*p* = 0.225, chi square test). A logistic regression analysis showed that only the Injury Severity Score was an independent risk factor (*p* = 0.014) for mortality, but not the type of therapy. This is summarized in Table 3.

**Table 3:** Logistic regression analyzing the risks for mortality. The only independent risk factor for mortality was a high ISS, but not the type of therapy or other epidemiological criteria

	OR	95% CI	P
Operative treatment	4.8 ± 7.3	0.25–93.01	0.295
ISS	0.89 ± 0.04	0.81–0.98	0.014
Age	0.96 ± 0.03	0.91–1.02	0.168
Maximal displacement	1.32 ± 0.48	0.64–2.71	0.456
Gender	1.67 ± 1.98	0.16–17.06	0.666

*ISS* Injury Severity Score, *CI* confidence interval, *OR* odds ratio

### Complications

9.4% (5/53) of operatively treated patients had complications, which was not statistically significant different from the 9.8% (6/61) observed in the group without operation (*p* = 0.471, chi square test). 5.6% of the operated patients had intraoperative complications, however, this did not increase the general complication rate, because all these patients suffered also from one of the general complications. The spectrum of complications in the operatively treated group covered besides the intraoperative complications, Adult Respiratory Distress Syndrome (ARDS), multi-organ failure, bleeding and infection, whereas the non-operatively treated patients suffered from pneumonia, sepsis, rhabdomyolysis, thrombosis and emboli. The average age of patients with complication was 56.5 years, females were overrepresented with 8 out of the 11 patients. The ISS ranged around the registered averages (18.7). None of the investigated parameters could be identified as an independent risk factor (Table 4).

**Table 4:** Logistic regression analyzing the risks for mortality. There is no independent risk factor for complications with statistical significance

	OR	95% CI	P
Operative treatment	1.26 ± 0.96	0.28–5.63	0.762
ISS	1.03 ± 0.04	0.96–1.11	0.342
Age	1.01 ± 0.01	0.98–1.04	0.634
Maximal displacement	0.96 ± 0.15	0.71–1.30	0.780
Gender	0.27 ± 0.20	0.06–1.18	0.082

*ISS* Injury Severity Score, *CI* confidence interval, *OR* odds ratio

### Influence of the anterior pelvic ring component

In order to analyze the influence of the anterior pelvic ring component, this injury was classified as follows: one-sided (*n* = 77, 67.5%) or two-sided injury (*n* = 37, 32.5%) of the anterior pelvis involving pubic and ischial ramus, butterfly injury (pubic and ischial ramus on both sides, *n* = 16, 14%) and non-butterfly injury (*n* = 98, 86%). Neither of these classifications were associated with more complications or a higher mortality (non-butterfly/ butterfly complications 9/89 vs 2/14, non-butterfly/ butterfly mortality 3/95 vs 2/14, one-sided/two-sided complications 8/69 vs 3/34, one-sided/two-sided mortality 3/74 vs 2/35).

### Follow-up

Considering the high effect of age on quality of life, patients of both groups were matched prior to a follow-up analysis for age and injury severity (ISS). By this, 10 patients of non-operatively and 9 patients of operatively treated patients could be recruited. The time to follow-up was 4.3 ± 2.0 [2–7] years. Table 5 summarizes the epidemiological data of these patients, showing no statistically significant difference considering all initially reported parameters.

**Table 5:** Overview about the epidemiological characteristics between matched non-operatively and operatively treated patients with lateral compression fractures of the pelvis, who had a follow-up examination

	non-operative	operative	P
Patients (n)	10	9	
Age (years)	45.5 ± 25.1	43.0 ± 22.5	0.823^b^
Gender (male/female)	5/5	2/7	0.210^a^
ISS	16.3 ± 8.8	19.9 ± 8.2	0.318^c^
Maximal displacement (mm)	2.2 ± 1.9	4.1 ± 2.1	0.051^d^
Length of hospital stay (days)	12.5 ± 7.4	16.0 ± 7.3	0.315^d^

*ISS* Injury Severity Score.

^a^chi square test; ^b^T-test; ^c^U-test (Mann-Whitney); ^d^Aspin-Welch T-test

As shown in Table 6, the Merle D’Aubigne and EQ. 5D-3 L scores were equally distributed in both groups. The VAS scores reported are part of the EQ. 5D questionnaires.

**Table 6:** Follow-up scores comparing matched non-operatively and operatively treated patients with lateral compression fractures of the pelvis

	non-operative	operative	P
Patients (n)	10	9	
EQ 5D-3 L	0.85 ± 0.14	0.88 ± 0.14	0.965^a^
VAS for general health status	7.7 ± 2.1	7.1 ± 2.0	0.508^a^
Merle d’Aubigne	16.3 ± 2.3	15.9 ± 2.2	0.768^a^

*VAS* Visual Analog Scale

^a^U-test (Mann-Whitney)

## Discussion

One of the main findings for this comparison of operative and non-operative treatment in lateral compression fractures of the pelvis was that we could determine the factors displacement, injury severity and age as crucial for decision-making. Although both groups varied a lot, there was no difference between the short-term outcomes in-hospital mortality and complication rate. Within this small cohort, only the injury severity was identified as an independent risk factor for mortality. For comparison of long-term follow-up, Merle D’Aubigne and EQ. 5D-3 L scores were evaluated in a matched subgroup, showing no difference between the treatment groups.

This data would indicate that neither short-term nor long-term results can be improved by surgical stabilization of type B2 fractures of the pelvis. However, this conclusion has a couple of limitations. Patients with injury characteristics usually requiring an operation such as disruption of the symphysis and complex injuries, including open fractures and injuries of internal organs, were excluded. Furthermore, the cohort represents only single-center data with a small number of patients. Despite, this is in accordance with other studies, which found no evidence that surgical stabilization of certain LC1 and LC2 pelvic fractures improved patients’ pain, decreased their use of analgesics, or improved time to mobilization [15]. All these factors are considered to be decisive for mobilization [14, 28]; and early physiotherapy is associated with less potential deadly complications. When looking a bit closer on the presented data for mortality, it becomes obvious that the rate ranges at the lower part of the expected interval, reflecting a rather stable injury and the exclusion of typically endangered patients. The mentioned bias of small numbers may be enhanced by this selection. Moreover, the relative frequency of dead was rather high in the conservatively treated group. A hidden possible positive effect of operative stabilization could be suspected as recently described in a much larger cohort [9], which, however, focused on both B- and C-type fractures. The only identified independent risk factor for mortality in this cohort of B2 fractures was injury severity, which is in line with previous publications on pelvic fractures in general [29–31]. Although we could identify parameters influencing decision-making for therapy, the presented data cannot answer, who should be operated, however, they are hypothesis generating. Why could it make sense to operate patients with a high ISS? They have other injuries besides a pelvic fracture, which is a problem with mobilization, especially when partial weight bearing is required. Fixation in this context provides more stability, resulting in less pain and increasing safety to prevent secondary displacement. To include maximal displacement of the posterior fracture component was proposed by several authors, suggesting that 5 mm might serve as a threshold for operative treatment [27]. Apparently, the degree of displacement somehow influences decision-making, however, no clear cut-off value could be identified. In contrast, considering the percentile analysis, there is a large overlap between operatively and conservatively treated patients. This can be explained by various reasons. The relevance is dependent on the location, where the displacement is measured. If the fracture is not complete, the significance is probably limited. Furthermore, CT scanning is only a snap-shot, giving no further information about the ligaments and the periosteum, which might be intact considering the injury mechanism of a compression. Furthermore, age was identified as being decisive for the choice of therapy. With increasing age and osteoporosis, fractures occur more frequently following low energy trauma, which is associated with less displacement and a lower ISS [32]. Therefore, this observation fits the conclusions made for the other criteria. A more pragmatic approach was recently suggested by Osterhoff et al., recommending operative stabilization only, if patients were not able to get mobilized [33]. This can certainly be very helpful, however, it is also related to uncertainty, because it lacks clear guidelines. A possible solution to solve this dilemma could be the use of standardized protocols such as the de Morton Mobility Index (DEMMI) [34] or the short physical performance battery (SPPB) [35]. Considering the design of this study, which is based on a register, this parameter cannot be evaluated.

The next research question of this study addressed, whether there is a difference regarding long-term outcome in the two differently treated cohorts. Acting on the assumption of comparable starting conditions except for type of therapy, there was no difference for quality of life and function. These results are in accordance with recently published data by Hagen et al. [15], showing also no evidence for improved patient’s functional outcome after surgical stabilization in lateral compression fractures of the pelvis.

A limitation of the study is the registry-based approach, which allowed to use only the items primarily indicated in the database. Therefore, the fracture classification comprised only the systematics provided by AO/OTA and not by Young Burgess, which specifically addresses the problems associated with lateral compression-type injuries of the pelvic ring. This applies also to the component of the anterior pelvic ring. Here, only the nature of the pelvic ramus fractures could be analyzed but not the actual displacement. However, it was a consensus decision of the steering committee to skip the absolute distances because of lacking relevance. Moreover, the mechanism of injury was not documented. The follow-up included only 19 patients, which might not be representative for the whole population. This was caused by the fact that most patients did not match the criteria for pairing. Furthermore, some patients were simply not available for follow-up.

## Conclusion

In conclusion, our study contributes to the debate about the necessity for surgical stabilization of type B2 fractures of the pelvis. Although in patients with low age, high injury severity and fracture displacement the surgeons’ decision favors operation, the type of therapy was not an independent risk factor for either in-hospital complications or mortality. Furthermore, operative stabilization does not seem to change the expected outcome. These results indicate that non-operative treatment might be sufficient, however, considering the study’s design the findings are only hypothesis generating and might support the design of future trials.

## Abbreviations

ARDS: Adult Respiratory Distress Syndrome
CI: confidence interval
eCRF: Case Report Form
EQ: EuroQuol
ISS: Injury Severity Score
LC: lateral compression
OR: Odds ratio
VAS: Visual Analog Scale
